# Codon usage, phylogeny and binding energy estimation predict the evolution of SARS-CoV-2

**DOI:** 10.1016/j.onehlt.2021.100352

**Published:** 2021-11-24

**Authors:** Matteo Calcagnile, Tiziano Verri, Maurizio Salvatore Tredici, Patricia Forgez, Marco Alifano, Pietro Alifano

**Affiliations:** a Department of Biological and Environmental Sciences and Technologies, University of Salento, Via Monteroni, 73100 Lecce, Italy; b INSERM UMR-S 1124 T3S, Eq 5 Cellular Homeostasis, Cancer and Therapy, University of Paris, Campus Saint Germain, Paris, France; c Thoracic Surgery Department, Cochin Hospital, APHP Centre, University of Paris, France; d INSERM U1138 Team «Cancer, Immune Control, and Escape», Cordeliers Research Center, University of Paris, France

**Keywords:** ACE2, ACE2 phylogeny, Animal host, B.1.1.7 (alpha) SARS-CoV-2 variant B.1.351 (Beta) SARS-CoV-2 variant, B.1.617.2 (delta) SARS-CoV-2 variant, Codon usage, Molecular evolution, Docking, SARS-CoV-2

## Abstract

In the frames of a One Health strategy, i.e. a strategy should be able to predict susceptibility to infection in both humans and animals, developing a SARS-CoV-2 mutation tracking system is a goal. We observed that the phylogenetic proximity of vertebrate ACE2 receptors does not affect the binding energy for the viral spike protein. However, all viral variants seem to bind ACE2 better in many animals than in humans. Moreover, two observations highlight that the evolution of the virus started at the beginning of 2020 and culminated with the appearance of the variants. First, codon usage analysis shows that the B.1.1.7 (alpha), B.1.351 (beta) and B.1.617.2 (delta) variants, similar in the use of codons, are also similar to a virus sampled in January 2020. Second, the host-specific D614G mutation becomes prevalent starting from March 2020. Overall, we show that SARS-CoV-2 undergoes a process of molecular evolution that begins with the optimization of codons followed by the functional optimization of the spike protein.

## Highlights

• The binding energy between the Spike protein and ACE2 (from animals) was calculated.
• There is no relationship between ACE2 protein phylogeny and spike binding.
• The mutation of SARS-CoV-2 on amino acid 614 reveals adaptation to humans.
• The codon usage is a good method to trace virus adaptation.
• B.1.1.7, B.1.351 and B.1.617.2 variants exhibit a similar codon usage caused by adaptation to humans.

## Graphical abstract

**Figure:**
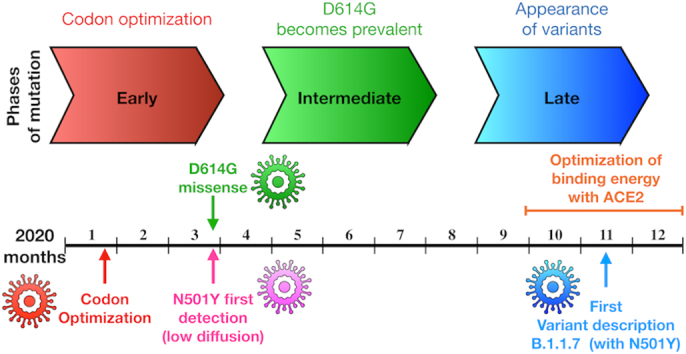

## 1 Introduction

A strategy to identify the host animal (‘reservoir’) of SARS-CoV-2 stands on the analysis of how the virus uses codons (‘codon usage’). The codon usage pattern of SARS-CoV-2 is similar to that of bat and human SARS-CoVs, although some (membrane protein-coding) genes are more similar to pangolin virus P1E [1], while others (orf1ab, spike, and nucleocapsid) show a codon usage pattern more similar to RaTG13 [1,2]. Spike and membrane protein-coding genes also undergo different evolutionary pressures [1]. Notably, the E gene of SARS-CoV-2 has the highest codon usage bias and appears to be under natural selection [3].

Most of the SARS-CoV-2 high-frequency codons end in A or T [2,4]. In addition, SARS-CoV-2 uses pyrimidine-rich codons more smoothly [4]. Also, SARS-CoV-2 has significantly lower GC compared to humans but, notably, its codon composition strongly correlates with that of some human genes expressed in the lung, suggesting that the GC content is crucial for adaptation to (a) specific host tissue(s) [4,5]. Adaptation to the human host is also supported by dinucleotide analysis: UG and CA dinucleotides are found to be preferred in SARS-CoV-2; in contrast, CG dinucleotide is avoided [6].

Besides similarity in codon usage, the binding energy between Receptor-Binding Domain (RBD) and Angiotensin-Converting Enzyme 2 (ACE2) also deserves attention. Computational methods have been used to estimate binding energy and similarity between ACE2 and RBD, from both human and vertebrates, with a focus on RaTG13 and Pangolin-CoV Spike proteins [[7], [8], [9]].

In these frames, we used a docking method whereby the N501Y mutation is predicted to increase ACE2 binding energy by ~ −20 Kcal/mol [10]. The prediction is now confirmed by both experimental and epidemiological data. In fact, N501Y influences protein S affinity for the receptor enhancing infectivity of the virus and, possibly, virulence [11,12], and all the most widespread variants in mid-2021 (June 2021), i.e., B.1.1.7 (alpha), B.1.351 (beta) and P.1 (gamma) show the N501Y mutation, despite their distinct geographical origin (United Kingdom, South Africa, and Brazil, respectively). However, at the end of 2021 (November 2021), the most common variant is B.1.617.2 (delta). This variant does not have the amino acid substitution N501Y but exhibits two other substitutions: L452R and T478K. As reported by an in vitro neutralizing assay performed with pseudo-typed viruses, L452R increases resistance to some monoclonal antibodies [13], while in-field observations suggest that administering one dose of the BNT162b2 vaccine produces 5.8 times lower neutralizing activity for variant B.1.617.2 (delta) than for variant B.1.1.7 (alpha). On the other hand, the administration of two doses restores the neutralizing effect [14]. Moreover, the two mutations also increase the binding affinity between ACE2 and RBD [15]. Following this line of reasoning, another mutation functionally emerges in B.1.617.2 (delta) variant, i.e. P681R, which instead of operating on ACE2 receptor binding seems to primarily act on S1/S2 cleavage by furin [16].

Another amino acid variation of the spike protein, which appeared in mid-2020 and then became prevalent, is D614G [17,18]. Present in B.1.1.7 (alpha), B.1.351 (beta), P.1 (gamma) and B.1.617.2 (delta) variants, it correlates with increased viral replication in the human lung epithelium, but it does not influence antibody-based neutralization [19,20].

In this study, we used codon analysis to compare human SARS-like (including SARS-CoV), and bovine, bat, and other mammalian viruses. The analysis included B.1.1.7 (alpha), B.1.351 (beta), and B.1.617.2 (delta) SARS-CoV-2. We used docking to investigate the relationships between phylogeny and the binding energy of RBD and ACE2. Docking simulations, initially performed using the RBD of SARS-CoV-2 (WT, Wuhan strain, S lineage), were extended using the RBD of the variant viruses B.1.1.7 (alpha), B.1.351 (beta), and B.1.617.2 (delta).

## 2 Methods

Sequences were downloaded from NCBI or UniProt. We used MODELLER v.10.1 to model ACE2 [21]. ACE2 N-terminal signal sequence was predicted using SignalP v.5 [22] and eliminated from the models. Phylogenetic analysis was performed using MEGA X [23], while we used ClustalO to align sequences [24]. We used HDOCK [25] to run the docking simulations, using ACE2 models (‘receptor’) and three different types of WT RBD (‘ligands’) (6 M17, 6LZG, and 6M0J). FireDock [26] was used to calculate the free binding energy (Kcal/mol) defined as Global Energy Score (GES). For each receptor, mean and standard deviation (SD) were calculated.

We performed a second set of simulation for the variant viruses. In this case we retrieved human RBD models from I-TASSER (B.1.1.7 and B.1.351) or RCSB (B.1.617.2, PDB ID: 7V7V). We modelled the interaction between ACE2 and RBD B.1.617.2 based on literature [15] (PDB ID: 6LZG).

We used the Sequence Manipulation Suite [27] to count the codons for SARS-CoV-2 viruses (WT and variants), and the Kazusa database (https://www.kazusa.or.jp/codon/) to count the codons of the other viruses. We used PAST to perform multivariate analysis for the codon usage data [28].

## 3 Results

### 3.1 ACE2 phylogenetic diversity in vertebrates and affinity to SARS-CoV-2 spike protein

**Fig. 1:**
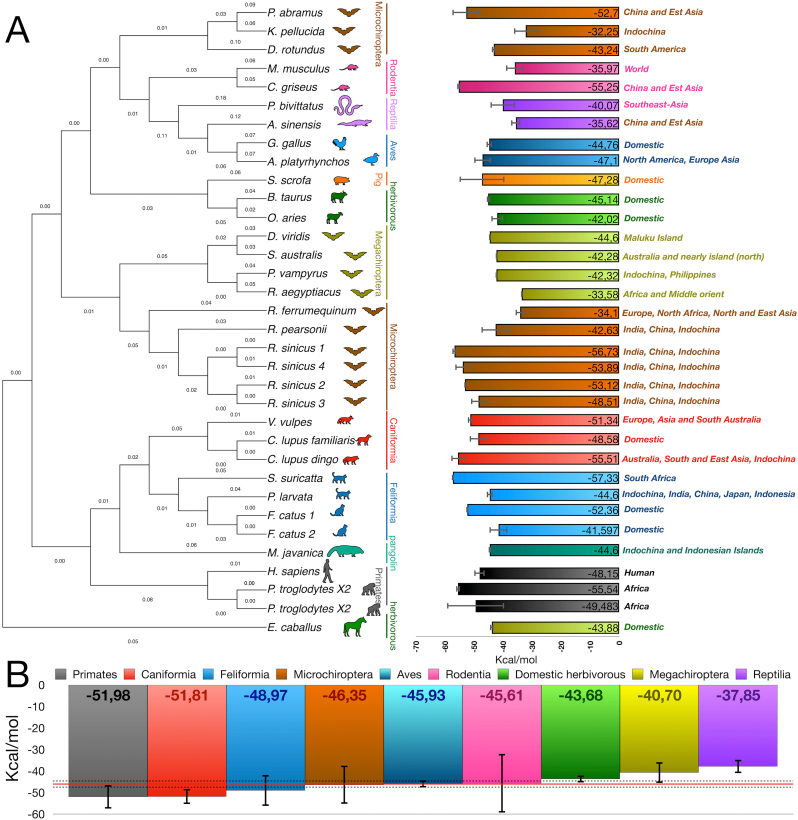
Binding energy estimation between ACE2 of vertebrates and RBD of SARS-CoV-2 and phylogenetic relationship between the ACE2 protein sequences. A) Phylogenetic tree showing the relationship between ACE2 of the selected vertebrates and binding energy between ACE2 proteins from vertebrates and RBD of SARS-CoV-2. B) Overall results obtained considering the reported class of vertebrates.

According to the docking results (Fig. 1, Tables S1 and S2), 13 out of 33 structures showed a better binding affinity with ACE2 proteins of vertebrates compared to the value obtained with human ACE2. The simulations included 2 proteins from *Pan troglodytes* (chimpanzee)*,* 5 out of 6 proteins from Carnivora and 5 out of 9 proteins from Microchiroptera. Primates showed better values, followed by Carnivora (Caniformia and Feliformia) and Microchiroptera (Fig. 1). Domestic herbivorous, Megachiroptera and Reptilia showed values significantly worse than the panel average.

**Fig. 2:**
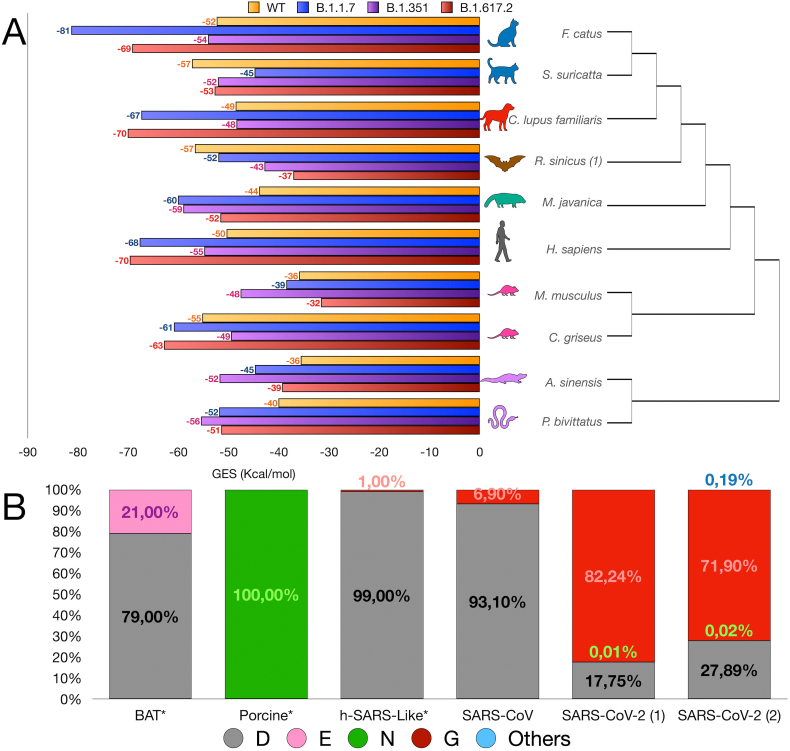
A) Binding energy estimation between vertebrate ACE2 and SARS-CoV-2 RBD (WT and variant virus B.1.1.7, B.1.351 and B.1.617.2). B) Diffusion of D614D/E/N/G substitution in bat, porcine and human coronavirus. (*) Sequences identified with BLAST.

The signal peptides also showed diversity in vertebrates. All sequences, but the reptilian *Python bivittatus* (Burmese python), exhibited a secretion signal (Fig. S1, Table S3). Two different cleavage sites were identified in many sequences: the cut occurring on S19 or A17 (ref. human ACE2). Fifteen sequences showed S19 as better cut site, while 9 sequences showed A17. All the sequences with a S19 cut site showed a conserved domain ‘LSLVAVTAAQS’, suggesting that modification of this motif can influence the cut. This domain was present also in 5 out of 15 proteins showing A17 as better site, but these 5 sequences showed a G in position 3, which can also influence the cut. According to this second set of docking simulations (Fig. 2A, Table S4), many species exhibited better GES values when the RBDs of variants B.1.1.7 (alpha) and B.1.351 (beta) were used. On the other hand, only 2 out of 10 [*Suricata suricatta* (meerkat)*, Rhinolophus sinicus* (Chinese rufous horseshoe bat) (1)] species exhibited worse values for B.1.1.7 and B.1.351 variants. The multiple alignments shown in Fig. S2 reveal that *S. suricatta* (meerkat)*, R. sinicus* (Chinese rufous horseshoe bat) share the N-terminal sequence of ACE2 that forms the most important interaction interface between RBD and ACE2. Hence, changes in these interfaces almost certainly result in a reduction in binding energy. As for the data generated with variant B.1.617.2 (delta), ACE2 sequences from *Felis catus* (cat)*, Canis lupus familiaris* (dog) and *C. griseus* (Chinese hamster) showed GES values comparable to that of human ACE2, while ACE2 sequences from all the other species showed less negative GES values. Specifically, *F. catus* (cat)*, C. lupus familiaris* (dog), *C. griseus* (Chinese hamster) and *H. sapiens* ACE2 sequences have very similar N-terminal sequences considering the first 120 amino acids (Fig. S2C).

### 3.2 The D614G mutation and its relationship with the reservoirs

The first noteworthy mutation that became prevalent as early as mid-2020 is D614G. This mutation is interesting because an acidic amino acid (D) is replaced by an apolar-achiral amino acid (G). We compared various CoVs of the porcine, bat, human, and SARS origin using BLAST. We also analyzed the databases reported in two studies [17,18] on SARS-CoV-2. Notably, D614G is recorded for SARS-CoV and other human CoVs since 2004 (Fig. 2B) [29]. Furthermore, a basic amino acid was identified (N) in the porcine CoV, while the bat viruses exhibited an acidic amino acid (D or E).

### 3.3 Codon analysis of the B.1.1.7 (alpha), B.1.351 (beta) and B.1.617.2 (delta) variants

The Relative Codon Frequency [RCF (%), File S1] was used to calculate Pearson's correlation index (RCF of human as reference) (Table S5). The values obtained indicate a positive correlation that changes depending on the virus group considered. Bovine viruses had low values (>0.17), while bat viruses had variable values (0.15–0.39). Of the bat viruses with higher values, 4 were CoV (HKU5-1, HKU5-2, HKU5-3 and HKU5-5) and 3 SARS-CoV (HKU3-1, HKU3-2, and HKU3-3). Human SARS-CoVs showed Pearson's indices ranging from 0.26 to 0.36, while civet SARS-CoVs exhibited high values (0.35 and 0.34), and the other two human CoVs exhibited very low values (0.18 and 0.06). Finally, the SARS-CoV-2 viruses exhibited values from 0.192 to 0.199 and, in addition, they exhibited a trend for the strains considered: the WT viruses showed values lower (generally around 0.192) than the B.1.1.7 (alpha) (around 0.198–0.199), B.1.351 (beta) (around 0.196–0.197) and B.1.617.2 (delta) (around 0.196–0.198) viruses.

**Fig. 3:**
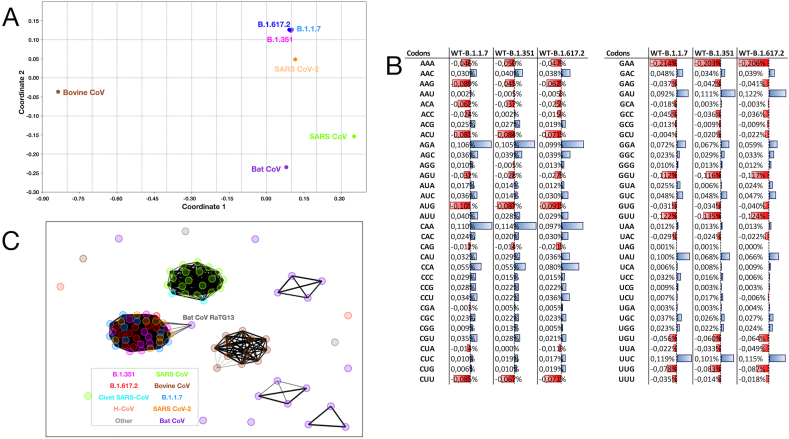
A) NM-MDS (ordination plot) showing the similarity and diversity between the codon usage of Bovine-CoVs, Bat-CoVs, SARS-CoV and SARS-CoV-2 (WT, B.1.1.7, B.1.351 and B.1.617.2). B) Variation of Relative Codon Frequency (RCF, %) calculated using the value of WT virus and subtracting the value of the variants (B.1.1.7, B.1.351 and B.1.617.2). C) Network plot showing the relationship between the viruses according to the data of the codon usage analysis.

Pearson's index was also calculated between mean RCF values obtained for 6 virus groups (B.1.1.7, B.1.351, B.1.617.2, Bat-CoV, Bovine-CoV, SARS-CoV) using as reference the values obtained for the SARS-CoV-2 WT (Table S5). Bovine CoVs exhibited the lowest value (0.89), SARS-CoV and Bat-CoVs exhibited similar values (0.952 and 0.955 respectively), and the three strains B.1.1.7 (alpha), B.1.351 (beta), B.1.351 and B.1.617.2 (delta) a value greater than 0.997. Fig. S3 shows the average values calculated for each codon for the groups SARS-CoV-2 (WT), B.1.1.7 (alpha), B.1.351 (beta), B.1.617.2 (delta), Bat-CoV, Bovine-CoV, SARS-CoV. Fig. S4 shows a violin plot considering all the databases and all the codons. The diversity of these groups of viruses is also shown by an NM-MDS using the Bray-Curtis index (Fig. 3A). Furthermore, the network plot (Fig. 3C) shows that Civet-CoV closely relates to SARS-CoV, while Bat-CoV RaTG13 closely relates to SARS-CoV-2. Two other network plots and an NM-MDS (Fig. S5) show the relationships between SARS-CoV-2 WT and the three variants B.1.1.7 (alpha), B.1.351 (beta), and B.1.617.2 (delta). This plot shows that the variants are similar one another and different from the SARS-CoV-2, although a WT virus is more like variants.

Comparing the values obtained by subtracting the RCF values of the B.1.1.7 (alpha), B.1.351 (beta), and B.1.617.2 (delta) viruses from the RCF calculated for the WT virus, it was evident that the RCF changes in a similar way for each codon (Fig. 3B). The Pearson's index calculated for the mean RCF values of the three viruses was >0.99.

## 4 Discussion and conclusions

The analysis of the use of codons has the advantage not only of considering both uncoded mutations and mutations that cause a change in the amino acid composition of proteins but also of providing information on the adaptation of the virus to the host. The codon usage bias was related to some specific features during the infection; e.g., it was observed that the infection with SARS-CoV-2 reduces the translation rate of highly expressed host proteins that share the codon usage bias of the virus [30]. Also, codon usage varied rapidly during the pandemic [3] as well as for isolates from distinct geographic regions [2]. Our results demonstrate that codon analysis is a valuable tool for tracking virus adaptation. Likewise, some specific mutations can be used for this purpose. The D614G is present in data referred to humans, for both SARS-CoV-2 and SARS-CoV. The same mutation is absent in bat, bovine and porcine viruses. This confirms D614G as a good marker to track viral evolution. The docking results reveal that carnivore ACE2 binds RBD with good energy. This confirms that some animals, including pets, can be hosts of SARS-CoV-2. Furthermore, the docking analysis for the variant form of the B.1.1.7 (alpha) and B.1.351 (beta) virus shows that the variants exhibit higher binding energy for almost all the animals considered. On the contrary, B.1.617.2 (delta) variant shows comparable binding energy values with ACE2 sequences from human, *F. catus* (cat)*, C. lupus familiaris* (dog) and *C. griseus* (Chinese hamster). These vertebrates also show a conserved N-terminal sequence suggesting that B.1.617.2 (delta) is well adapted to human ACE2. The docking data obtained in this study are consistent with the literature. E.g., the *Rhinolophus affinis* (intermediate horseshoe bat) ACE2 is a functional receptor for SARS-CoV-2 [31].

Taken together, these data show how computational approaches can be useful for analyzing and monitoring the spillover and evolution of viruses of environmental origin by comparatively analyzing sets of virus variants and vertebrates. Codon analysis is more effective in measuring genetic variations that occur over short periods. On the other hand, the docking analysis can provide information on animal hosts. In this respect, this work is a proof-of-concept, which can be useful for the implementation of bioinformatics and functional comparative genomics strategies from a One Health perspective. All the methods here shown are fast, low-cost, and powerful, which implies possible costs savings as a major benefit again from a One Health perspective. Last but not least, our approaches could be used to develop new protocols for rapid screening, screening of environmentally sourced viruses, bioinformatic tools to predict structure-to-function relationships, etc.

## Ethical approval

Not required.

## Author contributions

PA and MA conceptualized the study. MC performed computational analysis. TV implemented the database. PA, MA, and MC wrote the draft version. PF and MST revised the draft of the manuscript. All authors critically read and revised the manuscript and approved the manuscript for publication.

## Declaration of Competing Interest

All authors have no conflict of interest to declare.

## Appendix A Supplementary data

Complete database of codon usage data Image 1

Figure S1. Prediction of the signal of the secretion of ACE2. Figure S2. Multiple alignment of the N-Terminal ACE2 sequences. Figure S3. Average values of RCF (relative codon frequencies, %). Figure S4. Violin-Box plot of the whole database. Figure S5. NM-MDS and network plot of SARS-CoV-2 data.Table S1. Results of binding energy estimation between vertebrate ACE2 and SARS-CoV-2 RBD. Table S2. binding energy values for the groups of vertebrates (average value for each group). Table S3. Results of the prediction of the signal sequences in ACE2 used to perform docking. Table S4. Results of binding energy estimation between vertebrate ACE2 and SARS-CoV-2 RBD (WT, B.1.1.7, and B.1.351 and B.1.617.2). Table S5. Pearson’s index calculated considering human codon usage values as reference. Table S6. Location (country) and data of collection of SARS-CoV-2 sequenced genomes. Image 2
