# Supplementary material for: Codon usage, phylogeny and binding energy estimation predict the evolution of SARS-CoV-2
Source: One Health. 2021 Nov 24;13:100352. doi: 10.1016/j.onehlt.2021.100352 (PMC8610831; doi:10.1016/j.onehlt.2021.100352)
Supplement: Supplementary file 2 — Figure S1. Prediction of the signal of the secretion of ACE2. Figure S2. Multiple alignment of the N-Terminal ACE2 sequences. Figure S3. Average values of RCF (relative codon frequencies, %). Figure S4. Violin-Box plot of the whole database. Figure S5. NM-MDS and network plot of SARS-CoV-2 data.Table S1. Results of binding energy estimation between vertebrate ACE2 and SARS-CoV-2 RBD. Table S2. binding energy values for the groups of vertebrates (average value for each group). Table S3. Results of the prediction of the signal sequences in ACE2 used to perform docking. Table S4. Results of binding energy estimation between vertebrate ACE2 and SARS-CoV-2 RBD (WT, B.1.1.7, and B.1.351 and B.1.617.2). Table S5. Pearson’s index calculated considering human codon usage values as reference. Table S6. Location (country) and data of collection of SARS-CoV-2 sequenced genomes. [file mmc2.docx]

Pipistrellus abramus -------------------------MSSSSWLLLSLVAVAG**A**QYTTEEEARRFLVKFNHE 35

Kerivoula pellucida -------------------------MSGPTWLFLSLVAVAS**A**Q**S**LTEENARIFLQNFNSQ 35

Dobsonia viridis -------------------------MSGSFWLLLSLVAVTA**A**Q**S**TPEELVKTFLEKFNTE 35

Syconycteris australis -------------------------MSGSFWLLLSLVAVTA**A**Q**S**TPEELAKTFLEKFNAE 35

Pteropus vampyrus -------------------------MSGSFWLLLSLVAVTA**A**Q**S**TPEELAKTFLEKFNTE 35

Rousettus aegyptiacus -------------------------MSGSFWLFLSLVAVTA**A**Q**S**TPEELAKTFLEKFNTE 35

Desmodus rotundus -------------------------MSGSSWLFLSLVAVAA**A**QTPTEEEARTFLENFNTE 35

Sus scrofa -------------------------MSGSFWLLLSLIPVTA**A**Q**S**TTEELAKTFLEKFNLE 35

Bos taurus -------------------------MTGSFWLLLSLVAVTA**A**Q**S**TTEEQAKTFLEKFNHE 35

Ovis aries -------------------------MTGSFWLLLSLVAVTA**A**Q**S**TTEGQAKTFLEKFNHE 35

Mus musculus -------------------------MSSSSWLLLSLVAVTT**A**Q**S**LTEENAKTFLNNFNQE 35

Cricetulus griseus -------------------------MSSSSWLLLSLVAVTT**A**Q**S**IIEEQAKTFLDKFNQE 35

Rhinolophus ferrumequinum -------------------------MSGSSWLLLSLVAVTA**A**Q**S**TTEDLAKKFLDDFNSE 35

Rhinolophus pearsonii -------------------------MSGSFWFLLSLVAVTA**A**Q**S**TTEDRAKTFLDKFNHE 35

Rhinolophus sinicus (1) -------------------------MSGSFWLLLSLVAVTT**A**Q**S**TTEDRAKTFLDEFNSE 35

Rhinolophus sinicus (4) -------------------------MSSSSWLLLSLVAVTT**A**QFTTEDLAKIFLDEFNSE 35

Rhinolophus sinicus (2) -------------------------MSGSFWFLLSLVAVTT**A**Q**S**TTEDEAKIFLDKFNTK 35

Rhinolophus sinicus (3) -------------------------MSGSSWLLLSLVAVTT**A**Q**S**TTEDEAKMFLDKFNTK 35

Vulpes vulpes -------------------------MSGSSWLLLSLAALTA**A**Q**S**T-EDLVNTFLEKFNYE 34

Canis lupus familiaris -------------------------MSGSSWLLLSLAALTA**A**Q**S**T-EDLVKTFLEKFNYE 34

Canis lupus dingo -------------------------MSGSSWLLLSLAALTA**A**Q**S**T-EDLVKTFLEKFNYE 34

Suricata suricatta -------------------------MSGSFWLLLSFAALTA**A**Q**S**TTEELAKTFLEQFNHE 35

Paguma larvata -------------------------MSGSFWLLLSFAALTA**A**Q**S**TTEELAKTFLETFNYE 35

Felis catus -------------------------MSGSFWLLLSFAALTA**A**Q**S**TTEELAKTFLEKFNHE 35

Homo sapiens -------------------------MSSSSWLLLSLVAVTA**A**Q**S**TIEEQAKTFLDKFNHE 35

Pan troglodytes -------------------------MSGSSWLLLSLVAVTA**A**Q**S**TIEEQAKTFLDKFNHE 35

Equus caballus -------------------------MSGSSWLLLSLVAVTA**A**Q**S**TTEDLAKTFLEKFNSE 35

Manis javanica -------------------------MSGSSWLLLSLVAVTA**A**Q**S**TSDEEAKTFLEKFNSE 35

Python bivittatus MKQALIRKSSARSFTHPAFLDLKGNMLPWLCLIWSLVVLAVAQDVTQ-EAAEFLMQFDVR 59

Alligator sinensis -------------------------MLGYIWLFCGLFAV**A**V**P**QDVT-----TFLNQFNQN 30

Gallus gallus -------------------------MLLHFWLLCGLSAVVT**P**QDVTQ-EAQTFLAEFNVR 34

Anas platyrhynchos -------------------------MLAHVLLLCGLSTVVV**P**QDVTN-QAKMFLAEFNVR 34

**Red= best site**; **Blue= alternative site**  * :: .: :. * ** *:

**Figure S1.** Prediction of the signal of the secretion. The alignment shows the position of the cleavage site. The score of the best cleavage site was reported in the Table S3.


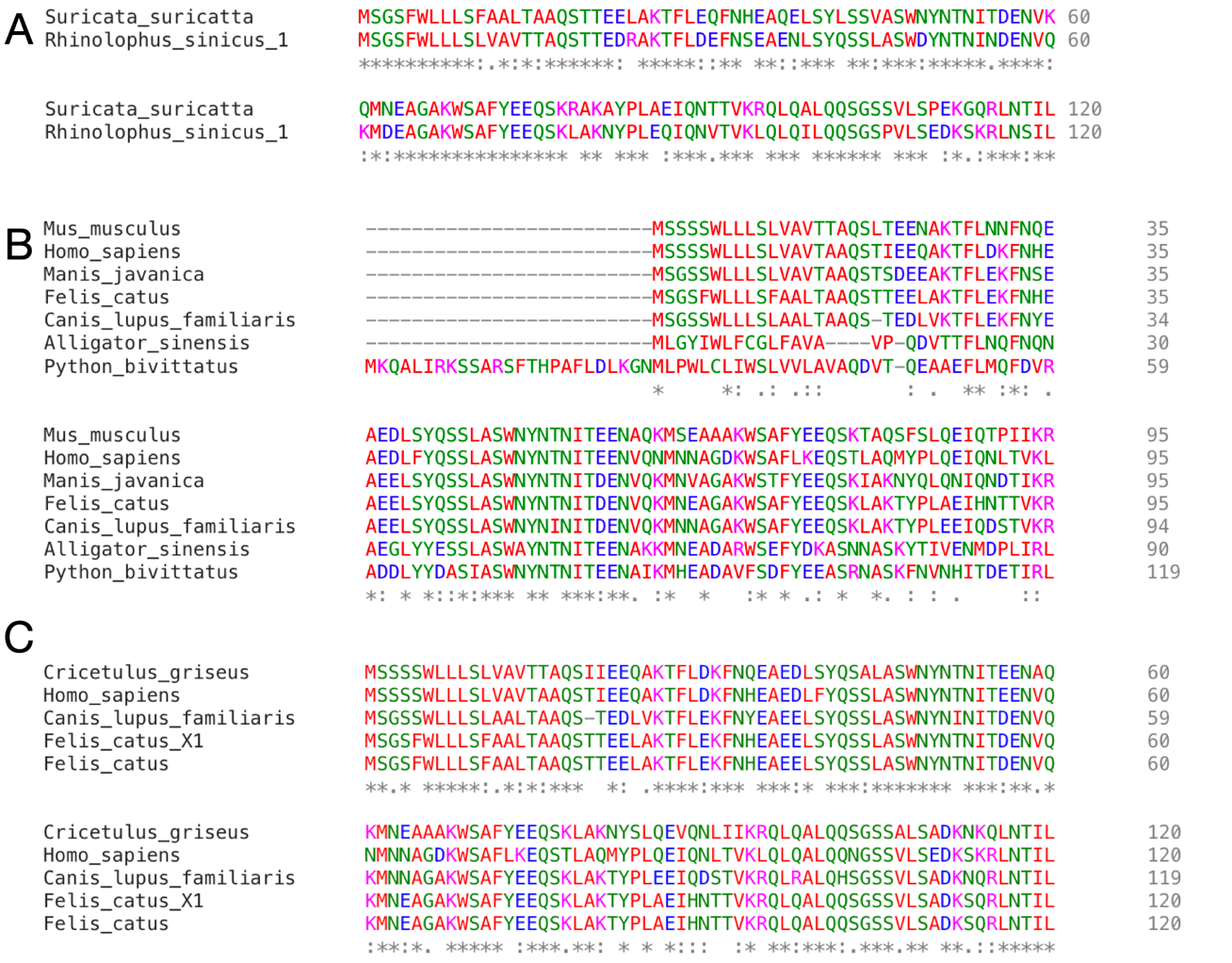


**Figure S2.** Multiple alignment of the N-Terminal ACE2 sequences reported in Fig. 2.

**Figure S3.** Average values of RCF (relative codon frequencies, %) calculated for each codon.


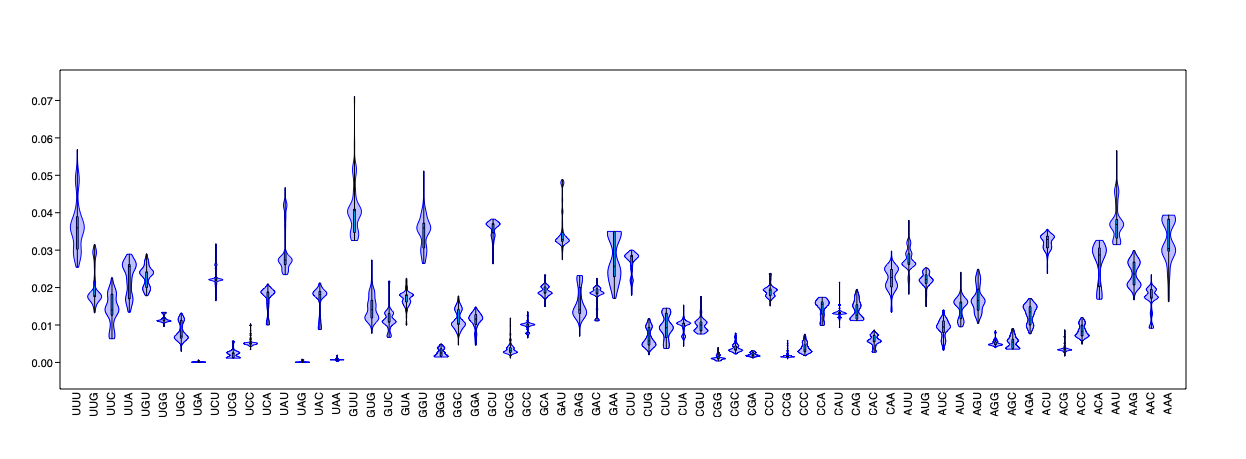


**Figure S4.** Violin-Box plot of the whole database. Whisker type= Standard error; Whisker length= One Sigma; Quartile method= Interpolation.

**Figure S5.** NM-MDS (A) (Bray-Curtis index) and network plot (cutoff=80%) (B) obtained using the codon usage data relative to SARS-CoV-2. Orange= SARS-CoV-2 WT; Blue= B.1.1.7; Fuchsia= B.1.351; Green=B.1.617.2. The sequence of SARS-CoV-2 WT that cluster with variant was sequenced in Australia (Melbourne) on 25 January 2020 (NCBI accession number MT007544.1).

**Table S1.** Results of binding energy estimation between vertebrate ACE2 and SARS-CoV-2 RBD. The docking simulations were performed using HDOCK, the binding energy (GES) was calculated with FireDock.

|  |  |  | **SARS-CoV-2 RBD** | | |  |  |
| --- | --- | --- | --- | --- | --- | --- | --- |
| **Genera species** | **Accession** | **Database** | **6LZG**^1^ | **6M0J**^1^ | **6M17**^1^ | **Average**^1^ | **SD**^2^ |
| H. sapiens | NP_001358344.1 | NCBI | -47.09 | -47.09 | -50.26 | -48.15 | 1.83 |
| P. troglodytes X2 | XP_016798469.1 | Uniprot | -55.43 | -55.43 | -56.54 | -55.8 | 0.64 |
| P. troglodytes X1 | XP_016798468.1 | NCBI | -43.79 | -43.79 | -60.87 | -49.483 | 9.86 |
| M. musculus | NP_081562.2 | Uniprot | -34.2 | -34.2 | -39.51 | -35.97 | 3.07 |
| C. griseus | XP_003503283.1 | NCBI | -54.91 | -54.91 | -55.94 | -55.25 | 0.59 |
| M. javanica | XP_017505746.1 | NCBI | -44.12 | -44.12 | -43.48 | -43.91 | 0.37 |
| P. larvata | AAX63775.1 | Uniprot | -43.99 | -43.99 | -45.82 | -44.6 | 1.06 |
| F. catus (1) | XP_023104564.1 | NCBI | -50.61 | -50.61 | -55.85 | -52.36 | 3.03 |
| F. catus (2) | NP_001034545.1 | NCBI | -39.82 | -39.82 | -45.15 | -41.597 | 3.08 |
| S. suricatta | XP_029786256.1 | NCBI | -57.11 | -57.11 | -57.76 | -57.33 | 0.38 |
| C. lupus familiaris | NP_001158732.1 | Uniprot | -46.91 | -46.91 | -51.92 | -48.58 | 2.89 |
| C. lupus dingo | XP_025292925.1 | NCBI | -56.85 | -56.85 | -52.83 | -55.51 | 2.32 |
| V. vulpes | XP_025842512.1 | Uniprot | -51.79 | -51.79 | -50.44 | -51.34 | 0.78 |
| S. scrofa | NP_001116542.1 | Uniprot | -42.83 | -42.83 | -56.18 | -47.28 | 7.71 |
| E. caballus | XP_001490241.1 | Uniprot | -43.49 | -43.49 | -44.67 | -43.88 | 0.68 |
| B. taurus | XP_005228485.1 | NCBI | -44.84 | -44.84 | -45.73 | -45.14 | 0.51 |
| O. aries | XP_011961657.1 | NCBI | -43.19 | -43.19 | -39.69 | -42.02 | 2.02 |
| R. sinicus **(1)** | ADN93475.1 | Uniprot | -56.31 | -56.31 | -57.57 | -56.73 | 0.73 |
| R. sinicus **(2)** | ADN93472.1 | Uniprot | -53.1 | -53.1 | -53.16 | -53.12 | 0.03 |
| R. sinicus **(4)** | ACT66275.1 | Uniprot | -55.4 | -55.4 | -50.87 | -53.89 | 2.62 |
| R. sinicus **(3)** | AGZ48803.1 | Uniprot | -49.99 | -49.99 | -45.56 | -48.51 | 2.56 |
| R. pearsonii **(1)** | ABU54053.1 | Uniprot | -39.86 | -39.86 | -48.17 | -42.63 | 4.8 |
| R. ferrumequinum | XP_032963186.1 | NCBI | -35 | -35 | -32.3 | -34.1 | 1.56 |
| P. vampirus | XP_011361275.1 | NCBI | -42.31 | -42.31 | -42.35 | -42.32 | 0.02 |
| R. aegyptiacus | XP_015974412.1 | NCBI | -33.57 | -33.57 | -33.59 | -33.58 | 0.01 |
| D. viridis | QJF77815.1 | NCBI | -44.6 | -44.6 | -44.6 | -44.6 | 0 |
| S. australis | QJF77811.1 | NCBI | -42.28 | -42.28 | -42.28 | -42.28 | 0 |
| G. gallus | XP_416822.2 | Uniprot | -44.23 | -44.23 | -45.82 | -44.76 | 0.92 |
| A. platyrhynchos | XP_012949915.2 | NCBI | -45.43 | -45.43 | -50.44 | -47.1 | 2.89 |
| A. sinensis | XP_025066628.1 | Uniprot | -34.68 | -34.68 | -37.49 | -35.62 | 1.62 |
| P. bivittatus | XP_007431942.2 | NCBI | -42.51 | -42.51 | -35.18 | -40.07 | 4.23 |
| D. rotundus | XP_024425698.1 | NCBI | -43.62 | -43.62 | -42.48 | -43.24 | 0.66 |
| K. pellucida | QJF77795.1 | NCBI | -34.57 | -34.57 | -27.6 | -32.25 | 4.02 |
| P. abramus | ACT66266.1 | NCBI | -55.44 | -55.44 | -47.22 | -52.7 | 4.75 |

^1^Binding energy was calculated as GES (Global Energy Score, Kcal/mol)

^2^ Standard Deviation

Median Value of all values= -46.05 Kcal/mol, SD of all values= 7.33, Dimension=102; confidence interval: ± 1.42

**Table S2.** GES values for the groups of vertebrates reported in the first column. The docking simulations were performed using HDOCK, the binding energy (GES) was calculated with FireDock.

| **Group** | **GES^1^** | **SD^2^** |
| --- | --- | --- |
| Primates | -51.97 | 5.41 |
| Microchiroptera | -46.35 | 8.86 |
| Carnivora (Feliformia) | -48.97 | 7.18 |
| Carnivora (Caniformia) | -51.81 | 3.49 |
| Rodentia | -45.61 | 13.63 |
| Domestic herbivorous | -43.68 | 1.57 |
| Megachiroptera | -40.69 | 4.87 |
| Aves | -45.93 | 1.65 |
| Reptilia | -37.84 | 3.15 |

^1^Binding energy was calculated as GES (Global Energy

Score, Kcal/mol)

^2^ Standard Deviation

**Table S3.** Results of the prediction of the signal sequences in ACE2 used to perform docking.

| **Vertebrates** | **Prediction** | **Cut site** | **Residues of cut** | **Probability** | **Likelihood** |
| --- | --- | --- | --- | --- | --- |
| Alligator sinensis | Signal peptide (Sec/SPI) | 15-16 | AVA-VP | 0.3754 | 0.9393 |
| Anas platyrhynchos | Signal peptide (Sec/SPI) | 17-18 | VVP-QD | 0.4962 | 0.7722 |
| Bos taurus | Signal peptide (Sec/SPI) | 17-18 | TAA-QS | 0.4163 | 0.9831 |
| Canis lupus familiaris | Signal peptide (Sec/SPI) | 17-18 | TAA-QS | 0.4284 | 0.9739 |
| Canis lupus dingo | Signal peptide (Sec/SPI) | 17-18 | TAA-QS | 0.4284 | 0.9739 |
| Cricetulus griseus | Signal peptide (Sec/SPI) | 19-20 | AQS-II | 0.4430 | 0.9426 |
| Desmodus rotundus | Signal peptide (Sec/SPI) | 17-18 | AAA-QT | 0.6598 | 0.9712 |
| Dobsonia viridis | Signal peptide (Sec/SPI) | 19-20 | AQS-TP | 0.3640 | 0.9873 |
| Equus caballus | Signal peptide (Sec/SPI) | 17-18 | TAA-QS | 0.3280 | 0.9647 |
| Felis catus | Signal peptide (Sec/SPI) | 17-18 | TAA-QS | 0.5232 | 0.974 |
| Gallus gallus | Signal peptide (Sec/SPI) | 17-18 | VTP-QD | 0.5541 | 0.9231 |
| Homo sapiens | Signal peptide (Sec/SPI) | 19-20 | AQS-TI | 0.3123 | 0.9504 |
| Kerivoula pellucida | Signal peptide (Sec/SPI) | 17-18 | ASA-QS | 0.5867 | 0.976 |
| Manis javanica | Signal peptide (Sec/SPI) | 19-20 | AQS-TS | 0.2813 | 0.9664 |
| Mus musculus | Signal peptide (Sec/SPI) | 19-20 | AQS-LT | 0.4872 | 0.9695 |
| Ovis aries | Signal peptide (Sec/SPI) | 17-18 | TAA-QS | 0.3450 | 0.984 |
| Paguma larvata | Signal peptide (Sec/SPI) | 17-18 | TAA-QS | 0.5204 | 0.9812 |
| Pan troglodytes | Signal peptide (Sec/SPI) | 17-18 | TAA-QS | 0.3345 | 0.9619 |
| Pipistrellus abramus | Signal peptide (Sec/SPI) | 17-18 | AGA-QY | 0.6773 | 0.9104 |
| Pteropus vampyrus | Signal peptide (Sec/SPI) | 19-20 | AQS-TP | 0.3602 | 0.9866 |
| Python bivittatus | Other |  | - |  | 0.6819 |
| Rhinolophus ferrumequinum | Signal peptide (Sec/SPI) | 17-18 | TAA-QS | 0.3370 | 0.9644 |
| Rhinolophus pearsonii | Signal peptide (Sec/SPI) | 17-18 | TAA-QS | 0.3710 | 0.9738 |
| Rhinolophus sinicus (1) | Signal peptide (Sec/SPI) | 19-20 | AQS-TT | 0.4060 | 0.9814 |
| Rhinolophus sinicus (2) | Signal peptide (Sec/SPI) | 19-20 | AQS-TT | 0.4400 | 0.9786 |
| Rhinolophus sinicus (3) | Signal peptide (Sec/SPI) | 19-20 | AQS-TT | 0.4613 | 0.9672 |
| Rhinolophus sinicus (4) | Signal peptide (Sec/SPI) | 17-18 | TTA-QF | 0.5223 | 0.8954 |
| Rousettus aegyptiacus | Signal peptide (Sec/SPI) | 19-20 | AQS-TP | 0.3626 | 0.9816 |
| Suricata suricatta | Signal peptide (Sec/SPI) | 17-18 | TAA-QS | 0.5254 | 0.9747 |
| Sus scrofa | Signal peptide (Sec/SPI) | 17-18 | TAA-QS | 0.4885 | 0.9618 |
| Syconycteris australis | Signal peptide (Sec/SPI) | 19-20 | AQS-TP | 0.3587 | 0.9837 |
| Vulpes vulpes | Signal peptide (Sec/SPI) | 17-18 | TAA-QS | 0.4242 | 0.9784 |

**Table S4.** Results of binding energy estimation between vertebrate ACE2 and SARS-CoV-2 RBD (WT, B.1.1.7, and B.1.351 and B.1.617.2).

|  | **GES^1^** | | | |
| --- | --- | --- | --- | --- |
|  | **WT** | **B.1.1.7** | **B.1.351** | **B.1.617.2** |
| *F. catus* | -52.36 | -81.35 | -54.1 | -69.22 |
| *S. suricatta* | -57.33 | -44.82 | -52.17 | -52.8 |
| *C. lupus familiaris* | -48.58 | -67.41 | -48.43 | -70.12 |
| *R. sinicus* (1) | -56.73 | -52.09 | -42.84 | -37.15 |
| *M. javanica* | -43.91 | -60.09 | -59.02 | -51.68 |
| *H. sapiens* | -50.45 | -67.74 | -54.83 | -45.03 |
| *M. musculus* | -35.97 | -38.55 | -47.61 | -31.61 |
| *C. griseus* | -55.25 | -60.87 | -49.47 | -62.87 |
| *A. sinensis* | -35.62 | -44.75 | -51.82 | -39.33 |
| *P. bivittatus* | -40.07 | -51.85 | -55.51 | -51.44 |

^1^Binding energy was calculated as GES (Global Energy

Score, Kcal/mol)

**Table S5.** Pearson’s index calculated considering human codon usage values as reference.

| **Virus** | **Pearson’s index** |
| --- | --- |
| **Feline CoV** | 0.242601836 |
| **Bat CoV RaTG13** | 0.197947058 |
| **Bat CoV HKU4-1** | 0.146245465 |
| **Bat CoV HKU4-2** | 0.146240435 |
| **Bat CoV HKU4-3** | 0.146117913 |
| **Bat CoV HKU4-4** | 0.146784376 |
| **Bat CoV HKU5-1** | 0.389494884 |
| **Bat CoV HKU5-2** | 0.377799281 |
| **Bat CoV HKU5-3** | 0.378098387 |
| **Bat CoV HKU5-5** | 0.378050551 |
| **Bat CoV HKU9-1** | 0.165661161 |
| **Bat CoV HKU9-2** | 0.192546388 |
| **Bat CoV HKU9-3** | 0.217493028 |
| **Bat CoV HKU9-4** | 0.228046862 |
| **Bat SARS CoV HKU3-1** | 0.357321192 |
| **Bat SARS CoV HKU3-2** | 0.357241503 |
| **Bat SARS CoV HKU3-3** | 0.357321192 |
| **Bat CoV** | 0.198649047 |
| **Bovine CoV** | 0.116555418 |
| **Bovine CoV R-AH65** | 0.167203373 |
| **Bovine CoV R-AH65-TC** | 0.167586125 |
| **Bovine CoV E-AH65** | 0.168684414 |
| **Bovine CoV E-AH65-TC** | 0.167036981 |
| **Bovine CoV R-AH187** | 0.167366131 |
| **Bovine CoV E-AH187** | 0.164801636 |
| **Bovine CoV DB2** | 0.168100808 |
| **Bovine CoV isolate Alpaca** | 0.185015356 |
| **Giraffe CoV US/OH3/2003** | 0.168314774 |
| **Calf-giraffe CoV US/OH3/2006** | 0.168314774 |
| **Giraffe CoV US/OH3-TC/2006** | 0.168232889 |
| **Porcine respiratory CoV** | 0.091276399 |
| **Sable antelope CoV US/OH1/2003** | 0.168172373 |
| **SARS CoV civet010** | 0.345828221 |
| **SARS CoV civet020** | 0.337906767 |
| **Human CoV 229E** | 0.18499867 |
| **Human COV NL63** | 0.058177913 |
| **SARS CoV** | 0.263430498 |
| **SARS CoV Tor2** | 0.343966096 |
| **SARS CoV Urbani** | 0.351375076 |
| **SARS CoV BJ01** | 0.363786999 |
| **SARS CoV CUHK-W1** | 0.3603963 |
| **SARS CoV CUHK-Su10** | 0.359244803 |
| **SARS CoV ZJ01** | 0.349671116 |
| **SARS CoV GD01** | 0.355318867 |
| **SARS CoV HSR 1** | 0.35552231 |
| **SARS CoV Taiwan TC1** | 0.352030675 |
| **SARS CoV Taiwan TC2** | 0.35126349 |
| **SARS CoV CUHK-AG01** | 0.359572221 |
| **SARS CoV CUHK-AG02** | 0.359572221 |
| **SARS CoV CUHK-AG03** | 0.359541893 |
| **SARS CoV Taiwan TC3** | 0.351853173 |
| **SARS CoV TWH** | 0.355414265 |
| **SARS CoV FRA** | 0.354732102 |
| **SARS COV NS-1** | 0.360063054 |
| **SARS CoV GZ02** | 0.340796996 |
| **SARS-CoV-2 (Wuhan) MT019532.1** | 0.192484866 |
| **SARS-CoV-2 (Wuhan) MT019533.1** | 0.192478323 |
| **SARS-CoV-2 (Wuhan) MT007544.1** | 0.19939502 |
| **SARS-CoV-2 (Wuhan) MN996527.1** | 0.194775007 |
| **SARS-CoV-2 (Wuhan) MN996528.1** | 0.194694162 |
| **SARS-CoV-2 (Wuhan) MT066176.1** | 0.192643312 |
| **SARS-CoV-2 (Wuhan) QHZ87581.1** | 0.192815077 |
| **SARS-CoV-2 (Wuhan) MT044258.1** | 0.192683554 |
| **SARS-CoV-2 (Wuhan) MT027063.1** | 0.192293696 |
| **SARS-CoV-2 (Wuhan) MT019530.1** | 0.192484866 |
| **SARS-CoV-2 (B.1.1.7) MZ201802.1** | 0.198263513 |
| **SARS-CoV-2 (B.1.1.7) MZ149972.1** | 0.199446753 |
| **SARS-CoV-2 (B.1.1.7) MZ201802.1** | 0.198263513 |
| **SARS-CoV-2 (B.1.1.7) MZ149898.1** | 0.199156282 |
| **SARS-CoV-2 (B.1.1.7) MZ149905.1** | 0.199670004 |
| **SARS-CoV-2 (B.1.1.7) MW531680.1** | 0.199076702 |
| **SARS-CoV-2 (B.1.1.7) MW856794.1** | 0.19983453 |
| **SARS-CoV-2 (B.1.1.7) MZ194585.1** | 0.198952047 |
| **SARS-CoV-2 (B.1.1.7) MZ195158.1** | 0.199457486 |
| **SARS-CoV-2 (B.1.1.7) MZ196334.1** | 0.198876253 |
| **SARS-CoV-2 (B.1.351) MW580244.1** | 0.196931488 |
| **SARS-CoV-2 (B.1.351) MW580574.1** | 0.197492738 |
| **SARS-CoV-2 (B.1.351) MW580576.1** | 0.197367556 |
| **SARS-CoV-2 (B.1.351) MW598408.1** | 0.197532335 |
| **SARS-CoV-2 (B.1.351) MW598413.1** | 0.197399375 |
| **SARS-CoV-2 (B.1.351) MW598419.1** | 0.197265333 |
| **SARS-CoV-2 (B.1.351) MW617734.1** | 0.19722032 |
| **SARS-CoV-2 (B.1.351) MW621453.1** | 0.197556569 |
| **SARS-CoV-2 (B.1.351) MW687146.1** | 0.196420102 |
| **SARS-CoV-2 (B.1.351) MW763124.1** | 0.196849617 |
| **SARS-CoV-2 (B.1.617.2) OL305594.1** | 0.19658365 |
| **SARS-CoV-2 (B.1.617.2) OK356418.1** | 0.19824739 |
| **SARS-CoV-2 (B.1.617.2) LC643036.1** | 0.19779996 |
| **SARS-CoV-2 (B.1.617.2) MZ397171.1** | 0.19762924 |
| **SARS-CoV-2 (B.1.617.2) MZ470855.1** | 0.1981568 |
| **SARS-CoV-2 (B.1.617.2) OK148246.1** | 0.19656986 |
| **SARS-CoV-2 (B.1.617.2) OK542667.1** | 0.19599548 |
| **SARS-CoV-2 (B.1.617.2) OK611832.1** | 0.19670781 |
| **SARS-CoV-2 (B.1.617.2) OL249797.1** | 0.19583135 |
| **SARS-CoV-2 (B.1.617.2) OL305519.1** | 0.19575616 |

**Table S6.** Location (country) and data of collection of SARS-CoV-2 sequenced genomes.

| **Strain** | **NCBI Accessionnumber** | **Collection date^1^** | **Country** |
| --- | --- | --- | --- |
| Wuhan | MT019532.1 | 30/12/19 | China, Beijing |
| Wuhan | MT019533.1 | 01/01/20 | China, Beijing |
| Wuhan | MT007544.1^*^ | 25/01/20 | Australia |
| Wuhan | MN996527.1 | 30/12/19 | China, Whuan |
| Wuhan | MN996528.1 | 30/12/19 | China, Whuan |
| Wuhan | MT066176.1 | 05/02/20 | Taiwan |
| Wuhan | QHZ87581.1 | 28/01/20 | USA |
| Wuhan | MT044258.1 | 27/01/20 | USA |
| Wuhan | MT027063.1 | 29/01/20 | USA |
| Wuhan | MT019530.1 | 30/12/19 | China, Whuan |
| B.1.1.7 | MZ201802.1 | 24/04/21 | USA |
| B.1.1.7 | MZ149972.1 | 30/01/21 | Togo |
| B.1.1.7 | MZ201802.1 | 24/04/21 | USA |
| B.1.1.7 | MZ149898.1 | 28/04/21 | USA |
| B.1.1.7 | MZ149905.1 | 28/04/21 | USA |
| B.1.1.7 | MW531680.1 | 01/06/21 | Bangladesh |
| B.1.1.7 | MW856794.1 | 01/06/21 | Hong Kong |
| B.1.1.7 | MZ194585.1 | 26/04/21 | USA |
| B.1.1.7 | MZ195158.1 | 01/05/21 | USA |
| B.1.1.7 | MZ196334.1 | 21/04/21 | USA |
| B.1.351 | MW580244.1 | 18/01/21 | France |
| B.1.351 | MW580574.1 | 29/01/21 | USA |
| B.1.351 | MW580576.1 | 01/02/21 | USA |
| B.1.351 | MW598408.1 | 10/01/21 | Ghana |
| B.1.351 | MW598413.1 | 12/01/21 | Ghana |
| B.1.351 | MW598419.1 | 14/01/21 | Ghana |
| B.1.351 | MW617734.1 | 27/01/21 | USA |
| B.1.351 | MW621453.1 | 03/01/21 | USA |
| B.1.351 | MW687146.1 | 20/02/21 | USA |
| B.1.351 | MW763124.1 | 20/02/21 | USA |
| B.1.617.2 | OL305594.1 | 14/08/21 | USA, Colorado |
| B.1.617.2 | OK356418.1 | 25/09/21 | India |
| B.1.617.2 | LC643036.1 | 06/21 | Japan, Tokyo |
| B.1.617.2 | MZ397171.1 | 28/05/21 | Myanmar |
| B.1.617.2 | MZ470855.1 | 20/06/21 | Bangladesh, Sylhet |
| B.1.617.2 | OK148246.1 | 06/09/21 | Bahrain |
| B.1.617.2 | OK542667.1 | 28/09/21 | USA, Florida |
| B.1.617.2 | OK611832.1 | 10/07/21 | USA, Maryland |
| B.1.617.2 | OL249797.1 | 08/09/21 | USA, Massachusetts |
| B.1.617.2 | OL305519.1 | 16/08/21 | USA: New York |

^*^ This virus cluster with variant viruses according to codon usage data

^1^ data was reported as day/month/year
